# Role of medial prefrontal cortex and primary somatosensory cortex in self and other-directed vicarious social touch: a TMS study

**DOI:** 10.1093/scan/nsad060

**Published:** 2023-10-14

**Authors:** Ashleigh Bellard, Paula D Trotter, Francis L McGlone, Valentina Cazzato

**Affiliations:** School of Psychology, Faculty of Health, Liverpool John Moores University, Liverpool, UK; School of Psychology, Faculty of Health, Liverpool John Moores University, Liverpool, UK; Institute of Psychology, Health & Society, University of Liverpool, Liverpool, UK; School of Psychology, Faculty of Health, Liverpool John Moores University, Liverpool, UK

**Keywords:** vicarious social touch, C-tactile afferents, social perception and cognition networks, transcranial magnetic stimulation, offline theta-burst stimulation

## Abstract

Conflicting evidence points to the contribution of several key nodes of the ‘social brain’ to the processing of both discriminatory and affective qualities of interpersonal touch. Whether the primary somatosensory cortex (S1) and the medial prefrontal cortex (mPFC), two brain areas vital for tactile mirroring and affective mentalizing, play a functional role in shared representations of C-tactile (CT) targeted affective touch is still a matter of debate. Here, we used offline continuous theta-burst transcranial magnetic stimulation (cTBS) to mPFC, S1 and vertex (control) prior to participants providing ratings of vicarious touch pleasantness for self and others delivered across several body sites at CT-targeted velocities. We found that S1-cTBS led to a significant increase in touch ratings to the self, with this effect being positively associated to levels of interoceptive awareness. Conversely, mPFC-cTBS reduced pleasantness ratings for touch to another person. These effects were not specific for CT-optimal (slow) stroking velocities, but rather they applied to all types of social touch. Overall, our findings challenge the causal role of the S1 and mPFC in vicarious affective touch and suggest that self- *vs* other-directed vicarious touch responses might crucially depend on the specific involvement of key social networks in gentle tactile interactions.

## Introduction

Interpersonal touch plays a pivotal role in non-verbal communication and is essential in the formation and maintenance of relationships (Morrison *et al.*, 2010; Brauer *et al.*, 2016; von Mohr *et al.*, 2017; Cascio *et al.*, 2019). Affective tactile experience during the very earliest stages of life is in fact deemed crucial for the development of the social brain (Cascio *et al.*, 2019). If not experienced, a lack of affective touch can have a negative long-lasting impact on the social brain, such as reduced grey matter and a reduction in brain activity (Nelson *et al.*, 2014).

Touch has been historically described as comprising of a discriminative/sensorimotor dimension, physiologically supported by myelinated Aβ afferent nerves, enabling fast conduction velocities and crucial for identifying external stimuli. This system ultimately allows rapid decision making which guides subsequent behaviour. Additionally, there is an affective dimension of touch, underpinned by specialized unmyelinated low threshold mechanosensory cutaneous C-tactile afferents (CTs), in the peripheral nervous system and predominately located in hairy skin (Liu *et al.*, 2007; McGlone *et al.*, 2014; Olausson *et al.*, 2010, but see Löken *et al.*, 2011; Watkins *et al.* (2021) for recent report of sparse innervation of CTs in the palm). CTs respond vigorously to gentle stroking of the skin, applied at velocities between 1 and 10 cm/s, with the greatest response occurring when touch is given at ∼3 cm/s at skin temperature (Löken *et al.*, 2009; Ackerley *et al.*, 2014); McGlone *et al.*, 2014). This type of touch is typically perceived as pleasant and rewarding in neurotypicals (Löken *et al.*, 2009; Ackerley *et al.*, 2014; Croy *et al.*, 2016) with CTs hypothesized to support the encoding of the hedonic value of interpersonal social touch (McGlone *et al.*, 2014).

Functional neuroimaging studies have offered insight into the neural pathways involved in touch processing, specifically those underpinned by the CTs fibres (Gordon *et al.*, 2013). These investigations have revealed the involvement of the posterior Insula Cortex in actual and anticipated experience of touch (Craig, 2002; Björnsdotter *et al.*, 2009; Gordon *et al.*, 2013; Lucas *et al.*, 2015; Morrison, 2016), a brain area which is understood to support the early convergence of sensory and affective signals about the body which in turn are then re-represented in the mid- and anterior portions of the insula, two brain sites which are responsible for the integration of interoceptive and contextual information (Critchley *et al.*, 2004; Craig, 2009; Evrard and Craig, 2015).

In addition to the insular cortex, previous neuroimaging studies have revealed that other key areas of the ‘social brain’ involved in social perception and social cognition (Gallagher and Frith, 2003; Olausson *et al.*, 2008; Morrison *et al.*, 2011; Gordon *et al.*, 2013; Koster-Hale and Saxe, 2013; Boehme *et al.*, 2019) are also responsible for the processing of the affective dimension of interpersonal touch. One of these brain regions includes the medial prefrontal cortex (mPFC) (Gordon *et al.*, 2013; Voos *et al.*, 2013; Chen *et al.*, 2020) which is well known for its involvement in theory of mind and mentalizing abilities (for reviews see Mar, 2011; Sperduti *et al.*, 2011) and is implicated in inferring other people’s intentions and mental states as well as attributing emotional states to others. Regarding affective touch, greater mPFC activation has been found previously when participants received manual brush stroking to the arm, compared to when they received brush stroking to the palm (Gordon *et al.*, 2013). Furthermore, a connectivity analysis using the mPFC as a seed region demonstrated that the insula and the amygdala are specifically involved in the processing of gentle touch delivered to the arm. Taken together these results suggest that the coactivation of the mPFC together with the amygdala and Insula during CT-optimal touch likely represents the encoding of social relevance and reward during the experience of CT-targeted affective touch (Gordon *et al.*, 2013) and strengthens the role of the skin as a ‘social organ’ (Morrison *et al.*, 2010).

Less conclusive evidence has been provided regarding the functional role of the primary somatosensory cortex (S1) in encoding the affective dimension of interpersonal touch. Whilst evidence supports the primary involvement of S1 in touch discrimination, including detection of tactile events (Cohen *et al.*, 1991), intensity and two-point discrimination of touch to the skin (Tegenthoff *et al.*, 2005), only recently findings suggest a putative involvement of S1 in understanding others’ sensations (Keysers *et al.*, 2004, 2010; Blakemore *et al.*, 2005; Ebisch *et al.*, 2008; Schaefer *et al.*, 2009, 2012; Pihko *et al.*, 2010; Bolognini *et al.*, 2013, 2014; Holle *et al.*, 2013; Kuehn *et al.*, 2013), a role which goes far beyond mere sensory discrimination. For instance, a study by Bolognini *et al.* (2013) which delivered low-frequency repetitive transcranial magnetic stimulation (rTMS) over S1 revealed that inhibition of S1 disrupted participants’ performance on a go/no-go task, but only when the affective state was conveyed by touch. Interestingly, this interfering effect was associated with individual differences in empathic ability to adopt the subjective perspective of others (but see Bowling and Banissy, 2017, for the lack of evidence of S1 modulation in vicarious tactile perception following high-frequency transcranial random noise stimulation). In a more recent TMS combined with electroencephalography study by the same group (Pisoni *et al.*, 2018), recordings were performed during tactile perception and observation to look for differences in cortical activation and connectivity between felt and seen touch. Findings from this study show that alpha connectivity within a frontoparietal pathway underpins the ability to distinguish self and others’ somatosensory states, controlling and distinguishing shared tactile representations in S1. Taken all together, these studies provide support that S1 could be endorsed with a dedicated tactile mirroring mechanism (Rizzolatti and Sinigaglia, 2016), allowing the automatic and unconscious simulation of others’ somatic states. Accordingly, this mirror activity of S1 may provide a neurophysiological substrate for matching inner self with other body representations and, in turn, an empathic interpersonal sharing of tactile events through the embodied simulation of the somatic sensations observed in others (Decety and Sommerville, 2003; Gallese, 2005; Grafton, 2009; Keysers *et al.*, 2010). Nevertheless, previous literature has not fully offered conclusive evidence for a strong link between S1 and social touch, specifically for the case of CT-targeted affective touch. One reason may be due to conflicting findings derived from the use of different behavioural paradigms and techniques, with only two neuromodulatory studies to date investigating the neural underpinnings of CT-optimal touch (Case *et al.*, 2016, 2017), with a specific focus on the role of S1. For instance, the studies by Case *et al.* (2016), Case *et al.* (2017) revealed that after participants received CT-optimal slow and non-CT-optimal fast gentle brushing of the hand proceeding rTMS over S1 (Case *et al.*, 2016) and over S2 (Case *et al.*, 2017), touch discrimination was reduced and rated as more intense, but pleasantness ratings remained unaffected. A further case-study conducted on a patient with acute polyradiculitis and polyneuropathy, i.e. loss of large-diameter myelinated afferents with a functioning CT-afferent system, still demonstrated typical pleasantness responses to receiving CT touch on hairy skin sites and only displayed deficits in their touch discrimination. Functional magnetic resonance imaging (fMRI) findings from this investigation revealed activation of the dorsal posterior insula cortex but not S1 (Olausson *et al.*, 2002). Taken all together, these findings cast doubts on whether affective touch is coded inside of S1, with the neural correlates involved in the control and distinction of shared tactile representations (within or outside S1) remaining largely unknown.

With these regard, it might be plausible that the existence of a shared tactile representation between perceived and observed touch may require some mechanisms subserving self-other distinction, allowing to code whom an activated tactile representation belongs to (i.e. self or other-directed touch). Therefore, with our current investigation, we aimed to understand whether S1 and mPFC are causatively involved in vicarious affective touch responses. Here, we took advantage of TMS, a non-invasive brain stimulation technique which enables the investigation of the causative role of a brain region in a specific behaviour, by inducing a temporary interference of neural activity. In creating this temporary interference, researchers can draw strong conclusions regarding whether the targeted brain region is necessarily involved with a specific function (Hallett, 2007). We therefore applied this approach to temporally perturb two crucial nodes of the social brain supposedly involved in the processing of shared representations of vicarious CT-targeted affective touch prior to participants observing an individual receiving touch at CT-optimal and CT non-optimal velocities (0, 5 and 30 cm/s), over several body regions (Ventral forearm, upper arm, back, cheek and palm). Importantly, we wanted to understand how an individual’s experience of touch might impact vicarious ratings of touch for self and others. Accordingly, tactile affective shared representations of vicarious gentle touch were investigated by two tasks which were designed to probe expectations of how touch is perceived by others (other-directed touch: How pleasant do you think the touch was for the person receiving it?) *vs* self (self-directed touch: How much would you like to be touched like this?). These two questions can also be considered as an implicit (other-directed) *vs* more explicit (self-directed) evaluation of pleasantness for CT-optimal touch, two dimensions of affective touch that can dissociate under certain circumstances. For example, two recent studies suggests that the vicarious experience of gentle touch is different in children in that they may not be able to detect a difference when the touch is delivered to another individual as compared to the self (Haggarty *et al.*, 2021a,b). More recently, Ali *et al.* (2023) reported that healthy participants’ ratings for how pleasant the touch was for the person receiving the touch in the video (other-focussed question) were significantly higher than ratings for how much they would like to be touched like that (self-focussed question). Finally, a further study on atypical vicarious affective touch demonstrated that current anorexics and remitted anorexics did not differ from the control subjects in their ability to rate touch to another person as a pleasant experience. However, when evaluating touch for themselves, they rated pleasant touching as being less enjoyable than the controls (Bellard *et al.*, 2022). Overall, results from these investigations open the questions as to whether different neurocognitive mechanisms may underlie subjective evaluations of vicarious social touch experiences for self and others.

Given that mPFC is greatly involved in affective mentalizing and in processing the rewarding value of CT-optimal touch, it is anticipated that interference with this region’s activity should result in reduced pleasantness ratings for CT-optimal touch compared to CT non-optimal touch when this is provided for others (other-directed touch). Furthermore, if according to Case *et al.* (2016), Case *et al.* (2017) encoding of the affective dimension of CT-optimal touch happens beyond S1, as well as the hedonic value of touch is intrinsically related to the physical characteristics of tactile stimuli, such as force, velocity, etc., then a temporary interference of S1 should result in reduced pleasantness ratings which should not be CT-optimal specific. Lastly, given that vicarious touch results in stronger behavioural responses when there is greater self-relatedness (Serino *et al.*, 2008, 2009; Cardini *et al.*, 2011, 2013), we expected S1 to be necessary for the visuo-tactile mirroring of touch and somatic experience related to touch for the self as opposed to others.

Finally, given that several studies have reported that neural responses to both experienced and seen touch vary in relation to several personality traits (Schaefer *et al.*, 2012; Voos *et al.*, 2013), in an explorative correlational analysis, top-down factors well known to influence touch responses, were also controlled for. Specifically, we focused on top-down factors, such as eating disorder symptomatology (Crucianelli *et al.*, 2016, 2019, 2021; Davidovic *et al.*, 2018; Cazzato *et al.*, 2021; Bellard *et al.*, 2022), interoceptive awareness (Adler and Gillmeister, 2019; Rigato *et al.*, 2019) and touch experiences and attitudes (Trotter *et al.*, 2018b), all of which are important for how an individual experiences social touch.

## Methods

### Participants

A total of 18 right-handed females aged 18–35 years (*M*_age_ = 23 yrs, s.d. = 4.26), were recruited and subject to all experimental conditions. The sample size required for our 3*3 (velocity*brain region) repeated-measures ANOVA design was determined using the G*power software (Faul *et al.*, 2009), setting expected effects size at 0.37 based on two previous non-invasive brain stimulation studies on vicarious affective touch (Peled-Avron *et al.*, 2019; Saporta *et al.*, 2022), *α*-level at 0.05, and desired power (1-*β*) at 95%.

The justification for only including females in this investigation is that recent studies have shown that females are more sensitive to affective touch, as well as to discriminative aspects of touch. Specifically, females rated affective touch and non-affective touch stimuli as more pleasant and had higher tactile acuity than males (Jönsson *et al.*, 2017). All participants were either students from Liverpool John Moores University or from the general public.

All participants had normal or corrected to normal vision (with glasses/contact lenses), no skin conditions, such as eczema, no chronic pain conditions, such as arthritis and had no past history of epilepsy or any form of neurological disease, no psychiatric conditions including a current or previous diagnosis of an eating disorder, did not have a cardiac pacemaker or any form of metal implants in the head and were not pregnant. All inclusion criteria were checked prior to testing to ensure participants met all inclusion criteria and those meeting the exclusion criteria did not participate. To ensure this and prior to testing, participants were administered with a TMS safety screening questionnaire to check for their eligibility to receive brain stimulation (Rossi *et al.*, 2011).

This investigation was conducted in accordance with the Helsinki declaration of ethical standards. The study protocol was approved by LJMU’s University Research Ethics Committee (UREC) (protocol: 21/PSY/002). All participants gave full informed consent to take part in the study and they were all debriefed at the end of the study. Participants were provided with a £15 amazon voucher and level 4 BSc Psychology students were awarded course credits, as compensation for their time.

### Demographics questionnaire

Demographic information that was taken from participants included their age, sex, gender, ethnicity and date of birth. Questions also asked participants to declare whether they have any skin conditions, such as psoriasis, eczema, etc. Height was collected by using a stadiometer and a calibrated bioimpedance digital scale (OMRON BF511) was used to measure participants’ body weight, for the calculation of participants’ body mass index (BMI).

### Self-report questionnaires

We controlled for possible confounding variables which are known to bias affective touch responses: eating disorder symptoms (Crucianelli *et al.*, 2016, 2019, 2021; Davidovic *et al.*, 2018; Bellard *et al.*, 2022), dysmorphic concerns and interoceptive awareness (Cazzato *et al.*, 2021), and touch experiences and attitudes (Devine *et al.*, 2020). Scores obtained by the EDI-3 and Dysmorphic Concern Questionnaire (DCQ) scales were compared to normative data to ensure comparability with the general population.

### Multidimensional Assessment of Interoceptive Awareness (MAIA)

The Multidimensional Assessment of Interoceptive Awareness (MAIA, Mehling *et al.*, 2012) is a 32‐item questionnaire which assesses eight components of interoceptive awareness: Noticing (4 items), Not Distracting (3 items), Not Worrying (3 items), Attention Regulation (7 items), Emotional Awareness (5 items), Self-regulation (4 items), Body Listening (3 items) and Trusting (3 items). Items were answered using a 6-point Likert scale ranging from 0 = Never to 5 = Always. Questions included: ‘When I am tense, I notice where the tension is located in my body.’ and ‘I notice when I am uncomfortable in my body’. Each individual dimension is scored by the average of scores from questions corresponding to that subscale, with some questions being reversed scored. This questionnaire has been previously used in neurophysiological research measuring associations between facets of metacognitive interoceptive and vicarious social touch (Adler and Gillmeister, 2019; Rigato *et al.*, 2019). The MAIA questionnaire was found to have good internal consistency, with Cronbach *α* = 0.90 (Valenzuela-Moguillansky and Reyes-Reyes, 2015). In this study, this was used to understand whether individual differences in metacognitive interoceptive awareness were associated with changes in pleasantness ratings consequent to continuous theta-burst transcranial magnetic stimulation (cTBS) over mPFC or S1.

### Eating Disorder Inventory-3 (EDI-3)

The Eating Disorder Inventory-3 (EDI-3) (Garner, 2004) is a 91 item self-report questionnaire assessing eating disorder symptomatology. This questionnaire assesses 12 subscales, 3 of which assess eating disorder symptomatology; Drive for Thinness, Bulimia and Body Dissatisfaction, which collectively examines eating disorder risk by summing these subscale scores (risk composite score). The other 9 subscales investigate personality traits generally associated with eating disorders: Low Self-esteem, Personal Alienation, Interpersonal Insecurity, Interpersonal Alienation, Interoceptive Deficit, Emotional Dysregulation, Perfectionism, Ascetism and Maturity Fear. Questions are answered using a 6-point Likert scale ranging from 0 = never to 5 = always. This questionnaire has been previously validated with clinical and non-clinical samples across various cultures (Clausen *et al.*, 2011). Also, individual subscales, such as Interoceptive Deficits, better described as a measure of emotional awareness, was also used to assess one’s ability to understand and recognize internal bodily sensations and emotional states (Garner, 2004). This questionnaire has good internal consistency in clinical populations, with Cronbach *α* = 0.80–0.92 (Clausen *et al.*, 2011) and in healthy populations with Cronbach *α* = 0.78–0.93 (Garner, 2004; Clausen *et al.*, 2011). In this study, this measure was used to control whether ED symptomatology was associated with changes in touch pleasantness ratings consequent to TMS disruption to mPFC or S1.

### Dysmorphic Concern Questionnaire (DCQ)

The DCQ (Oosthuizen *et al.*, 1998) is a short 7-item questionnaire and is a reliable tool which assesses both behavioural and cognitive aspects of dysmorphic concern. Each item is rated using a 4-point Likert scale ranging from 0 = not at all, 4 = much more than most people. All 7 items were totalled for each participant to give an overall score for dysmorphic concern. These scores range from a minimum of 0 to a maximum of 21, with a score of 9 or more being indicative of high dysmorphic concern (Mancuso *et al.*, 2010). The DCQ has good internal consistency with Cronbach *α* = 0.80 (Jorgensen *et al.*, 2001). This measure was used to determine whether levels of concerns towards physical appearance were associated with changes in pleasantness ratings after TMS disruption to mPFC or S1.

### Touch Experiences and Attitudes Questionnaire (TEAQ)

The Touch Experiences and Attitudes Questionnaire (TEAQ, Trotter *et al.*, 2018b) is a 57-item questionnaire which was administered to examine current experiences of positive touch and positive experience of touch during childhood, as well as an individual’s attitude towards positive touch. Questions were answered using a 5-point Likert scale ranging from 1 = ‘Disagree strongly’, 2 = ‘Disagree a little’, 3 = ‘Neither agree nor disagree’, 4 = ‘Agree a little’, 5 = ‘Agree strongly’. Questions included: ‘I dislike people being very physically affectionate towards me.’ and ‘There was a lot of physical affection during my childhood.’ A mean score was calculated for each of the six subscales; friends and family touch (11 items), current intimate touch (14 items), childhood touch (9 items), attitude to self-care (5 items), attitude to intimate touch (13 items) and attitude to unfamiliar touch (5 items), with negatively worded questions reversed scored. The TEAQ questionnaire was found to have good internal consistency with Cronbach *α* = 0.78–0.92 (Trotter *et al.*, 2018b). This questionnaire assessed whether changes in pleasantness of touch consequent to inhibition of mPFC or S1 were associated with experiences and attitudes of touch.

## Measures

### Tactile estimation task

Given the involvement of S1 in the intensity of touch (Case *et al.*, 2016) and the processing of two-point discrimination of touch to the skin (Tegenthoff *et al.*, 2005), we administered the tactile estimation task (TET) to rule out any tactile disturbances from participants which may be causative of a reduction in S1 activation. In doing so, this eliminates the chances of any false claims associated with a reduction in pleasantness ratings from cTBS-S1, rather than due to tactile disturbances (Goodin *et al.*, 2018).

The TET has also been previously used with the general and Anorexia Nervosa (AN) populations (Keizer *et al.*, 2011, 2012; Zopf *et al.*, 2021). The TET for this investigation involved applying two tactile stimuli simultaneously to the right forearm of the participants prior to any brain stimulation. Participants were asked to wear clothing that allowed easy access to their forearm.

During this task, participants were provided with a blindfold and asked to estimate the distance between the two tactile stimuli using their thumb and index finger and place their fingers onto the whiteboard provided. The distance between the thumb and index finger was then measured by the researcher using a ruler and for each trail, the distance was noted. The distance the tactile points on the calliper was placed on the participants forearm differed, i.e. 50, 60 and 70 mm measurements were used. These measurements were applied randomly to the same body region to prevent participants using previous measurements to guide their estimation of the same measurement, which in turn could result in order effects making them more accurate (Keizer *et al.*, 2011). To ensure participants did not experience any discomfort during this task, only female researchers were present during this part of testing (and during all testing).

### Affective touch video clips

This task was displayed using E-prime 2.0 software (Psychology Software Tools, Pittsburgh, PA). The observed affective touch task consisted of 6-s touch videos of a male applying touch to various body areas of a female actress. Touch was provided across five different body regions, which included a glabrous skin site with little CT innervation, the palm and four hairy skin sites with greater CT innervation: the ventral forearm, upper arm, cheek and back. Touch was provided at three different velocities [static (0 cm/s), slow (5 cm/s) and fast (30 cm/s)] for each body region.

The order in which the videos were viewed was fully randomized amongst participants. After viewing each video, participants were probed to respond to one of two questions: ‘How pleasant do you think that action was for the person being touched?’ (other-directed touch) using a VAS scale ranging from 0 = ‘very unpleasant’ to 100 = ‘extremely pleasant’ and ‘How much would you like to be touched like that?’ (self-directed touch) using a VAS scale ranging from 0 = ‘not at all’ to 100 = ‘extremely’ (Walker *et al.*, 2017; Bellard *et al.*, 2022).

**Fig. 1.**
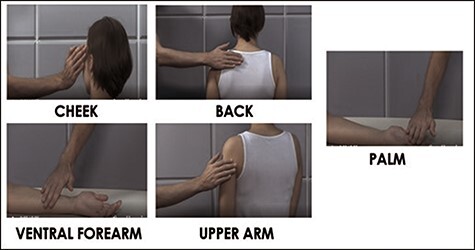
Visual illustration of the five body sites (CT-innervated body regions: ventral forearm, upper arm, cheek and back *vs* the non-CT innervated palm) from the affective touch videos used for the two self- and other-directed touch tasks in this study.

Both tasks were blocked, so participants only answered one question per block and blocks were counterbalanced. For each block, there was a total of 45 videos displayed, as each touch video was presented three times per block in a randomized order. There were two blocks presented, one for each condition (self-directed touch and other-directed touch) which were displayed once for each of the three brain regions (mPFC, S1 and vertex), with a total of six blocks. Overall, across all conditions and blocks, there was a total of 270 videos presented, each displayed in 240 p YouTube quality (Trotter *et al.*, 2018a) (see Figure 1).

### TMS

The experiment involved three visits to the lab, in which participants were subject to all brain stimulation conditions which were counterbalanced amongst participants. All participants were subject to three offline rTMS with theta-burst protocol sessions which were delivered over S1, mPFC and vertex (control region) on the right hemisphere, with one brain region targeted per session. TMS sessions lasted 40 s (200 bursts, each comprising three pulses at 50% power, 30 Hz frequency, 6 Hz burst frequency repeated every 200 ms (5 Hz), 600 pulses in total) as detailed in Goldsworthy *et al.* (2012). This occurred prior to the presentation of the observed affective touch task. As a result, participants were required to attend three lab sessions, to prevent any confound of previous stimulation interfering with results from another brain region (Avenanti *et al.*, 2012; Ding *et al.*, 2014; Pozdniakov *et al.*, 2021). Participants received this protocol to the right S1, mPFC and vertex using a 70 mm figure-of-eight stimulation coil (Magstim Double 70 mm Air Film Coil and D70 Air Film Coil), connected to a Magstim SuperRapid^2^ Stimulator (The Magstim Company, Carmarthenshire, Wales), this generated a magnetic field up to 0.8 T at the surface of the coil.

**Fig. 2.**
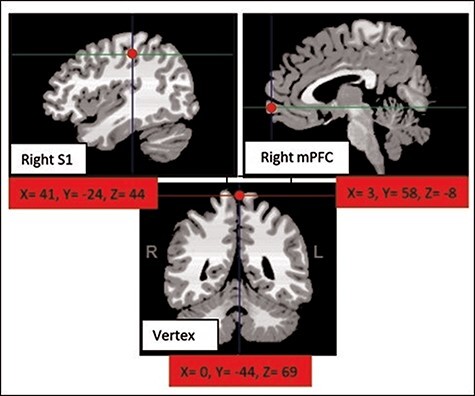
Visual depiction of the location of each of the three brain regions on the right hemisphere: medial prefrontal cortex (mPFC), primary somatosensory (S1) and vertex, as shown in MRIcro template. The red dots indicate the position the coil was placed on participants’ scalp. All coordinates are converted to Talairach.

Prior to the brain stimulation phase, right S1, mPFC and vertex target regions were localized by means of stereotaxic navigation on individual estimated magnetic resonance images (MRIs) obtained through a 3D warping procedure fitting a high-resolution MRI template with the Davidovic *et al*. (2019) participant’s scalp model and craniometric points (Softaxic 3.0, EMS, obtained using individual MRI scans, see Carducci and Brusco, 2012). Repetitive TMS with a theta-burst protocol was delivered over the right S1 (*X* = 46, *Y* = −28, *Z* = 72) following the localization of the same brain area by Case *et al.* (2016). mPFC coordinates were located based on a previous study by Davidovic *et al*. (2019) using coordinates (*X* = 3, *Y* = 58, *Z*  = −8) and specifically from a main contrast showing which brain regions were more active when participants received stroking (compared to vibration); following this study, we targeted the mPFC located close to the Brodmann’s Area 10 in the anterior mPFC. As a control site, the vertex was localized as the point falling half the distance between the nasion and the inion on the same midline and was stimulated with the induced current running from posterior to anterior along the interhemispheric fissure (*X* = 0, *Y* = − 44, *Z* = 69) (Cazzato *et al.*, 2014) (see Figure 2).

### General procedure

Interested participants were sent the screening questionnaire and TMS safety-screening questionnaire via email and asked to fill it in and send it back to be checked before any testing sessions were booked in. The TMS safety-screening assessed participant’s eligibility to safely receive brain stimulation and the screening questionnaire assessed standard criteria for the study, such as being female, over 18, no chronic pain, skin conditions, etc. Those participants who were eligible, based on responses from the TMS safety screening questionnaire and screening questionnaire were contacted by the researcher to arrange three testing sessions, each with a minimum of 48 h in between.

For session 1, lasting ∼1 h, participants were asked to re-complete the TMS safety screening questionnaire, to ensure no changes have occurred since completing it online. Participants were then asked to complete the demographics questionnaire, EDI-3, MAIA, DCQ and TEAQ. Once completed, participants were asked to sit comfortably and complete the TET. This task involved applying two tactile stimuli simultaneously to the forearm. Participants were blindfolded and asked to estimate the distance between the two tactile stimuli placed at various distances, using their thumb and index finger.

Proceeding from this, the Softaxic Neuronavigation system was used to create a 3D reconstruction of the participant’s brain using the nasion, Inion, A1 and A2 as well as localizing 19 individual points on the scalp. This 3D reconstruction was used to localize the first brain region, by entering Talairach Co-ordinates for that specific brain region. This 3D brain reconstruction was saved using a unique participant code, to be used for future sessions. After a reconstruction was created, participants received offline TMS with a theta-burst protocol, in which 600 pulses were provided for a duration of 40 s to one of the three brain regions (mPFC, S1 and vertex), depending on counterbalancing order. To successfully target the mPFC and for the coil to remain in place in this region, participants were seated in a chair which was laid flat when this stimulation was provided. Once stimulation was finished, participants immediately completed the two observed affective touch tasks in two separate blocks. This required participants to view videos of a male actor applying touch to a female across five body sites (ventral forearm, upper arm, cheek, back and palm) with three different velocities [static (0 cm/s), slow (5 cm/s) and fast (30 cm/s)]. After viewing each video, in two separate blocks each corresponding to a question, participants were asked to respond: ‘How pleasant do you think that action was for the person being touched?’ (other-directed touch) using a VAS scale ranging from 0 = very unpleasant to 100 = extremely pleasant and ‘How much would you like to be touched like that?’ (self-directed touch) using a VAS scale ranging from 0 = not at all to 100 = extremely (Walker *et al.*, 2017). The same procedure was repeated for sessions 2 and 3, each one lasting ∼20 min. Finally, participants were debriefed and provided with a full account of what the study was about and the hypothesis of the investigation. Overall, the study lasted a maximum of 2 h.

### Statistical analysis and data processing

All demographic information and scores for the self-reported questionnaires are reported as mean (M) and s.d. in Table 1.

**Table 1.** Range scores for demographics, self-report questionnaires scores and TET estimations for all participants (n=18). The column on the far right displays the mean and standard deviation (in brackets)

	Range	Mean (s.d.)
Age (years)	18.00–35.00	22.00 (4.26)
BMI (kg/cm^2^)	20.52–36.73	24.19 (4.64)
EDI-3		
Drive for thinness	0.00–20.00	7.67 (5.59)
Bulimia	1.00–30.00	12.06 (9.85)
Body dissatisfaction	0.00–32.00	9.56 (11.67)
Low self-esteem	0.00–16.00	6.61 (5.46)
Personal alienation	0.00–14.00	5.39 (4.39)
Interpersonal insecurity	0.00–13.00	5.33 (4.03)
Interpersonal alienation	1.00–16.00	4.50 (3.65)
Interoceptive deficit	0.00–26.00	5.94 (6.28)
Emotional dysregulation	0.00–14.00	4.33 (3.40)
Perfectionism	0.00–21.00	7.67 (5.40)
Ascetism	0.00–14.00	4.11 (3.72)
Maturity fear	0.00–20.00	8.11 (5.36)
Composite score	4.00–59.00	29.28 (18.70)
DCQ (max 21)	1.00–15.00	6.83 (4.20)
MAIA		
Noticing (max 5)	1.00–4.00	2.78 (0.87)
Not distracting (max 5)	0.00–3.67	2.35 (0.93)
Not worrying (max 5)	0.33–4.00	2.18 (1.03)
Attention regulation (max 5)	0.43–3.71	2.24 (0.83)
Emotional awareness (max 5)	0.80–4.00	2.84 (0.90)
Self-regulation (max 5)	0.75–4.00	2.33 (0.79)
Body listening (max 5)	0.00–3.33	1.65 (1.02)
Trusting (max 5)	0.67–3.67	2.22 (0.70)
TEAQ		
Friends and family touch (max 5)	2.18–4.64	3.57 (0.68)
Current intimate touch (max 5)	2.43–4.64	3.78 (0.68)
Childhood touch (max 5)	2.67–5.00	3.99 (0.76)
Attitude to self-care (max 5)	3.00–5.00	3.97 (0.75)
Attitude to intimate touch (max 5)	2.00–5.00	4.06 (0.81)
Attitude to unfamiliar touch (max 5)	2.00–4.00	2.87 (0.71)
Tactile estimation task (TET)		
Baseline	25.00–45.00	34.17 (6.47)
50 mm	38.60–103.80	58.67 (17.24)
60 mm	33.00–131.40	65.02 (22.97)
70 mm	23.60–139.40	72.11 (27.97)
Total	38.47–124.87	64.93 (20.50)

*BMI* body mass index*; EDI-3* eating disorder inventory; *DCQ* dysmorphic concern questionnaire; *MAIA* multidimensional assessment of interoceptive awareness; *TEAQ* touch experiences and attitudes questionnaire; *s.d.* standard deviation

All statistical analyses were implemented in STATISTICA version 8.0 (StatSoft, Tulsa, OK, USA) and/or IBM SPSS (Statistics version 26). Given the fact that null hypothesis significance testing is the main statistical method in neuroscience, we first used frequentist ANOVAs to show the effect of cTBS over the three regions of interest on vicarious ratings of touch. Accordingly, a two-way ANOVA with within-subject factors of velocity (0, 5 and 30 cm/s) and brain region (mPFC, S1 and vertex) was conducted separately for each task (self-directed touch and other-directed touch). *Post hoc* pairwise comparisons were carried out using the Newman–Keuls test. The *α* value for all statistical tests was set at 0.05. Effect sizes were obtained using the partial *η*-squared. However, null hypothesis significance testing cannot assess whether observed data favour the null hypothesis in comparison to the alternative hypothesis, which in our study is critical to determine whether cTBS manipulations were ineffective in changing vicarious ratings of self- and other-directed touch. Therefore, we complemented ANOVAs with their Bayesian implementations using JASP (JASP Team, 2022, v0.16.3). By doing so, we directly evaluated the relative strength of evidence for the null and alternative hypotheses, providing quantification of the degree to which the data support either hypothesis (Dienes, 2011; Wagenmakers *et al.*, 2018; Keysers *et al.*, 2020). Default priors in JASP were used. Inclusion Bayes Factors (BFs) quantify the evidence for including a specific main effect or interaction. A BF >3 indicates evidence for the alternative hypothesis, whereas a BF <0.3 indicates evidence for the null hypothesis (Jeffreys, 1961). A BF between 0.3 and 3 indicates an inconclusive result which is not in favour of either hypothesis.

Finally, for each task, we also performed Pearson’s correlations (Bonferroni-corrected, *α*/5, *P* = 0.01) as well as their Bayesian implementation, considering the index Δ(Brain Areas X − Vertex) for touch ratings with scores obtained for the EDI-3, MAIA, TEAQ, DCQ questionnaires and TET. This allowed us to account for the potential contribution of confounding variables, such as body image disturbances and social touch attitudes and experiences to the experimental findings.

## Results

### Univariate statistics

Table 1 demonstrates the ranges, mean and s.d. for the demographics, self-report questionnaire scores and TET estimations for all participants. Participants in this sample had an average healthy (fell into the normal weight classification following WHO categories) BMI score. Scores obtained for the EDI-3 and DCQ were compared to normative data to ensure comparability with the general population. In keeping with Clausen *et al.* (2011), EDI-3 Interoceptive deficit subscale scores for the current study were indicative of the general population (5.94 ± 5.50; *t*(17) = 0.300, *P* = 0.768). In keeping with Mancuso *et al.* (2010), scores for the DCQ varied from no dysmorphic concern to great dysmorphic concern, with the average being indicative of low dysmorphic concern towards one’s body (6.83 ± 4.46; *t*(17) = 2.395, *P *= 0.028).

Table 2 demonstrates the average (*x, y, z*) Talairach coordinates averaged across all participants for the mPFC, S1 and vertex, which were the average coordinates used to localize the coil onto the scalp. All coordinates have been taken from that reported from the SofTaxic Neuronavigation system. All coordinates are originally reported in MNI space and have been converted to Talairach.

**Table 2.** Demonstrates the Talairach coordinates averaged across all participants (n= 18) for each brain region (mPFC, S1, and Vertex) for the localisation of the coil using the Softaxic Neuronavigation system

	X	Y	Z
Brain regions			
mPFC	5.86	56.71	−10.57
S1	45.57	−27.71	71.86
Vertex	1.43	−42.14	68.43

*mPFC* medial prefrontal cortex; *S1* primary somatosensory cortex

### Vicarious ratings of self-directed touch

The two-way within-subjects ANOVA of brain region (mPFC, S1 and vertex) × Velocity (0, 5 and 30 cm/s) for touch ratings for oneself, revealed a significant main effect of brain region [*F*_(2,34)_ = 3.770, *P = *0.033, *ηp^2^* =0.182, *BF_incl_ *= 1.938]. Significantly higher pleasantness ratings were provided for S1-cTBS (51.55 ± 3.84) compared to vertex-cTBS (45.80 ± 3.06, *P* *=* 0.037, *BF_10_ *= 26.046) and mPFC-cTBS (46.87 ± 3.28, *P* *=* 0.043, *BF_10_* = 2.79). No evidence for a significant difference between mPFC-cTBS and the vertex-cTBS was observed (*P* = 0.635, *BF_10_* = 0.188).

**Fig. 3.**
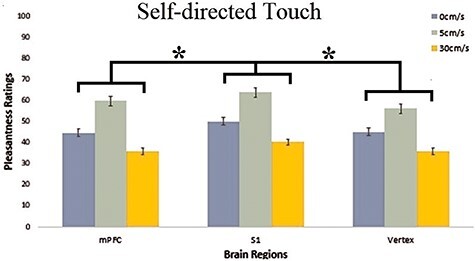
Pleasantness (VAS) ratings for each CT-optimal and non-optimal velocity (0, 5 and 30 cm/s) for each of the 3 brain regions (mPFC, S1 and vertex) for self-directed touch. cTBS over S1 selectively increased touch willingness for touch to self, compared to inhibition of mPFC and vertex. This effect was not specific for touch delivered at CT-optimal velocities. Error bars indicate SEM over participants *P < 0.05.

There was also strong evidence for a significant main effect of velocity [*F*_(2,34)_ = 15.408, *P *< 0.001, *ηp^2^* = 0.48, *BF_incl_ *= 1116.5]. Touch to the self was greater for touch velocities of 5 cm/s (60.05 ± 5.13) compared to touch delivered at 0 cm/s (46.69 ± 2.84, *P *< 0.001, *BF_10_ *= 14 606.3) and 30 cm/s velocities (37.48 ± 3.50, *P *< 0.001, *BF_10_ * = 1.035 × 10^+8^). Furthermore, touch delivered at 0 cm/s velocity (46.69 ± 2.84) received greater ratings compared to that delivered at 30 cm/s (37.48 ± 3.50, *P* *=* 0.031, *BF_10_ *= 61.176). Surprisingly, there was no significant two-way interaction between brain region × velocity [*F*_(4,68)_  = 0.503, *P *= 0.733, *ηp^2^* = 0.03, *BF_incl_ *= 0.083], with the Bayes factor analysis providing stronger evidence for no interaction between the two factors (see Figure 3 for a visual breakdown of results).

**Fig. 4.**
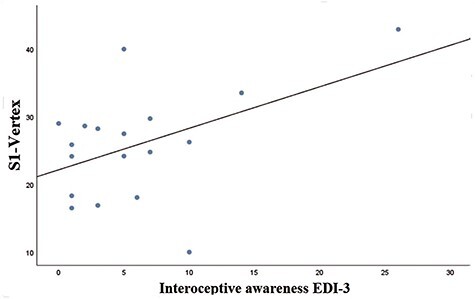
Correlation between Δ[S1-Vertex] index and interoceptive awareness (EDI-3) for self-directed touch. Individuals with greater interoceptive (emotional) awareness reported greater desire to receive social touch (regardless of CT-optimal velocities) after cTBS over S1 (compared to the vertex).

We then conducted Pearson’s Correlational analyses for self-directed touch considering the index Δ[S1-Vertex] for touch ratings with EDI-3, MAIA, TEAQ, DCQ questionnaires and TET to understand if any effects from non-invasive brain stimulation on pleasantness ratings was associated with any of the scales. Results showed evidence for a significant positive correlation between emotional awareness (interoceptive deficit subscale from EDI-3) and change scores after S1-cTBS (*r* = 0.604, *P* = 0.008, *BF_10_ *= 7.642, see Figure 4). Thus, participants with higher levels of emotional awareness rated they would like to receive touch more following inhibitory cTBS stimulation of the S1 than following vertex stimulation. No other correlations were significant (all *r*s > −0.295, all *P*s > 0.959, see *Supplementary Materials* for a full report of the results obtained by these correlations).

In summary, regardless of cTBS to brain regions, our data provide strong evidence that CT-optimal velocity of 5 cm/s was always preferred when asked about touch to self, compared to CT non-optimal velocities, i.e. 0 and 30 cm/s. Crucially, cTBS over S1 increased ratings for touch to self, compared to inhibition of mPFC and vertex (although Bayes Factor analysis suggested that compared to mPFC-cTBS, this effect was statistically inconclusive and therefore it remains unclear whether the effect of S1-cTBS was location-specific). Interestingly, this finding was also associated with evidence of a greater levels of emotional awareness (EDI-3). Finally, the increase in self-directed touch ratings after S1-cTBS was not CT-optimal touch specific, as also demonstrated by the Bayesian statistics which showed evidence for the absence of a selective effect of stimulation after cTBS, specifically for CT-optimal (slow) affective touch.

### Vicarious ratings of other-directed touch

The two-way within-subjects ANOVA of brain region (mPFC, S1 and vertex) × Velocity (0, 5 and 30 cm/s) for touch ratings for another, revealed a significant main effect of brain region [*F*_(2,34)_ = 4.384, *P = *0.020, *ηp^2^* =0.205, *BF_incl_ *= 1.479]. cTBS-mPFC (46.92 ± 2.32) significantly lowered pleasantness ratings compared to cTBS-vertex (51.33 ± 2.72, *P = *0.026, *BF_10_ *= 13.923) and cTBS-S1 (50.70 ± 2.04, *P* = 0.025, *BF_10_ *= 5.255). Importantly, cTBS over S1 (50.70 ± 2.04) did not significantly lower pleasantness ratings compared to vertex-cTBS (51.33 ± 2.72, *P* = 0.701, *BF_10_ *= 0.163).

**Fig. 5.**
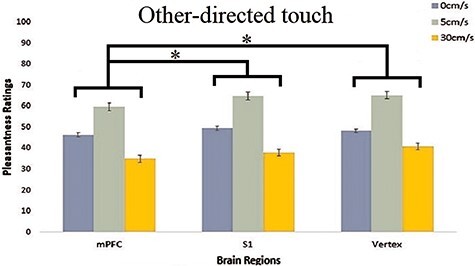
Pleasantness (VAS) ratings for each CT-optimal and non-optimal velocity (0, 5 and 30 cm/s) for each of the 3 brain regions (mPFC, S1 and vertex) for other-directed touch. cTBS over mPFC selectively decreased touch pleasantness for touch to other, compared to inhibition of mPFC and vertex. This effect was not specific for touch delivered at CT-optimal velocities. Error bars indicate SEM over participants *P < 0.05.

There was also strong evidence for a significant main effect of velocity [*F*_(2,34)_ = 22.803, *P *< 0.001, *ηp^2^* = 0.573, *BF_10_ *= 19 758.1]. Other-directed touch delivered at 5 cm/s (63.17 ± 4.06) was rated as significantly more pleasant compared to touch delivered at 0 cm/s (47.96 ± 2.02, *P* < 0.001, *BF_10_ *= 4.505 × 10^+6^) and at 30 cm/s (37.82 ± 2.84, *P* < 0.001, *BF_10_ *= 7.463 × 10^+9^). Furthermore, touch delivered to others at 0 cm/s (47.96 ± 2.02) was rated as significantly more pleasant compared to touch delivered at 30 cm/s (37.82 ± 2.84, *P*= 0.011, *BF_10_ *= 750.011). Similar to results obtained for the self-directed touch task, there was no significant two-way interaction between brain region × velocity [*F*_(4, 68)_= 0.768, *P *= 0.550, *ηp^2^* = 0.04, *BF_10_ *= 0.324, with the Bayes factor analysis providing strong evidence in favour of no interaction between the two factors (see Figure 5).

Pearson’s correlational analyses for other-directed touch separately considering the index Δ[mPFC-Vertex] for pleasantness ratings with variables from EDI-3, MAIA, TEAQ, DCQ questionnaires and TET scores revealed no significant correlations with Δ[mPFC-Vertex] and any subscales (all *r*s > −0.397, all *P*s >0.102, see *Supplementary Materials* for a full report of the results obtained by these correlations).

In summary, our data provide strong evidence that overall, other-directed touch delivered at a CT-optimal velocity of 5 cm/s was always preferred compared to non-optimal velocities of 0 and 30 cm/s. cTBS delivered over the mPFC specifically decreased pleasantness ratings compared to cTBS delivered over vertex and S1, whilst evidence for a lack of significant difference was provided when comparing S1- to vertex-cTBS. Like the result obtained for self-directed touch, the lack of a significant two-way interaction of brain region and velocities suggests that the effects of stimulation over mPFC are not specific for CT-optimal (slow) touch. This result was also confirmed by the Bayesian statistics which showed evidence for the absence of a selective interferential effect of cTBS, specifically for CT-optimal (slow) affective touch.

## Discussion

To the best of our knowledge, this was the first study to investigate whether perturbing the mPFC and S1 by means of cTBS is causative in altering ratings for self- and for other-directed vicarious CT-targeted affective touch. A point of novelty was that we investigated the neural bases of vicarious CT-optimal touch by focusing not only on the shared tactile representation of the participant with the touch receiver when touch is directed to self, but also on the tactile representation of the participant with another person, in S1 and mPFC. To this aim, we employed a neuromodulatory technique, cTBS, to gain insight into the causal involvement of S1 and mPFC, two brain areas supposedly necessary for the encoding of affective and sensory dimensions of vicarious CT-optimal touch. We also controlled for confounding variables that have been shown to impact touch responses, such as eating disorder symptoms (Crucianelli *et al.*, 2016, 2019, 2021; Davidovic *et al.*, 2018; Bellard *et al.*, 2022), dysmorphic concerns (Cazzato *et al.*, 2021), interoceptive awareness (Adler and Gillmeister, 2019; Rigato *et al.*, 2019) and touch experiences and attitudes towards touch (Trotter *et al.*, 2018b; Devine *et al.*, 2020).

Our findings showed that cTBS stimulation of S1, compared to the vertex, resulted in participants reporting greater ratings for self-directed touch, thus suggesting a key role of S1 in the visual processing of self-directed touch (regardless of CT touch optimality). On the other hand, based on the Bayes Factor analysis, it remains unclear whether this increase in ratings for self-directed touch after S1-cTBS, compared to mPFC-cTBS, is location-specific. If there is an effect, it is relatively small.

Our findings resonate with previous research evidence that right S1 is functionally involved in visuo-tactile mirroring mechanisms important for evaluating our experience of touch, based on the observation of another being touched (Keysers *et al.*, 2004; Blakemore *et al.*, 2005). Accordingly, we speculate that a shared tactile representation in S1 of observed somatic feelings due to resonance mechanisms may allow the interpretation (re-mapping) of others’ tactile events for self (Keysers *et al.*, 2004; Blakemore *et al.*, 2005; Bufalari *et al.*, 2007; Ebisch *et al.*, 2008; Schaefer *et al.*, 2009; Pihko *et al.*, 2010; Wood *et al.*, 2010; Adler *et al.*, 2016; Deschrijver *et al.*, 2016). Nevertheless, according to this reasoning, it could be expected that upon inhibition of S1 it would be no longer possible to experience the positive, rewarding value of self-directed touch, which in turn should lead to a decrease rather than to an increase in the liking to be touched. Our findings could be explained within the predictive coding framework (Friston and Stephan, 2007, 2008; Huang and Rao, 2011). Accordingly, perception of another person receiving touch is dependent upon noise of incoming sensory signals to constantly generate and update a mental model of this action (Beal, 2003). The brain acts as a predictive machine and uses this generated model to make predictions of sensory input and compare this to incoming actual sensory signals, with the main purpose of minimizing prediction errors—the difference between predictions and the actual signal (Huang and Rao, 2011). The brain then forms Bayesian-optimal predictions (i.e. apply probabilities) of future scenarios which must be constantly revised, and prior beliefs updated through the input of new sensory information. Therefore, if such principles apply to the inhibition of S1 in the current study, it may be plausible to think that by adding noise into the somatosensory signal, this in turn could have led to greater prediction errors as these incoming signals would have been classified as unreliable. As a result, the brain may use prior beliefs, i.e. crucially that participants know this is a ‘pleasant’ (non-painful) touch experience so have greater willingness to be touched in the same way, rather than using belief updating and make the decision that they know the touch is pleasant and want to be touch more like it. A further alternative explanation, more in line with the results obtained by Case *et al.* (2016) which shows increased ratings of brushing intensity after inhibitory TMS to S1, could be related to the fact that in our study, cTBS over S1 might have caused a reduced sensory discrimination, perhaps of intensity. Therefore, future studies should focus on assessing changes in tactile sensation of intensity during observed CT-targeted affective touch.

Notably, perturbation of S1 did not result in a reduction in touch ratings for the self when touch was delivered at CT-optimal (i.e. slow) stroking velocities, given that this effect was also observable in non-CT optimal touch. Accordingly, the Bayes Factor analysis provides evidence for the lack of interaction between cTBS effects and CT-optimal velocities, which may speak in favour of the fact that rather than playing a role in visuo-tactile mirroring specifically for CT-targeted (slow) affective touch, S1 may be more involved in the processing of all forms of affective touch (both delivered at CT-optimal and non-CT optimal speeds). These findings are also in line with the linear positive association observed between changes in liking to be touched upon S1-cTBS and self-reports of emotional awareness (as measured by the EDI-3 scale). Accordingly, for self-directed touch, after cTBS-S1, we observed that the higher the liking to be touched, the higher the levels of emotional awareness. This finding is not in fact surprising. Emotional awareness is an essential process for human psychosomatic health, with disturbance of this type of awareness leading to unhealthy conditions through obstruction of homeostatic processing (Kanbara and Fukunaga, 2016). However, it should be noted that this measure of Interoceptive deficits (EDI-3) is limited in its assessment of true interoception, that is the distinction between somatic, as opposed to emotional awareness (Eshkevari *et al.*, 2014). It is important therefore for future investigations to evaluate interoceptive awareness using other self-report measures that more directly assess somatic awareness, as opposed to emotional awareness. Nevertheless, we cannot provide any conclusive evidence of correlations between facets of metacognitive interoception as measured by MAIA and changes of ratings for self-directed touch following S1-cTBS. With these regard, a recent study by Adler and Gillmeister (2019) found that individuals with better interoceptive abilities, specifically the ability to sustain and control attention to bodily signals, also have stronger vicarious representations of observed touch within somatosensory cortices. Bodily and emotional awareness is an increasing research field (e.g. Khalsa *et al.*, 2018), and the investigation of potential relationships with vicarious representations of interpersonal touch is likely to be advanced through the development of more refined neuromarkers of implicit interoception (e.g. heartbeat evoked potentials, Schulz *et al.*, 2015).

When looking at ratings for other-directed touch, a further novel result of our study was that inhibition of the mPFC caused a reduction in pleasantness ratings when making inferences regarding someone else receiving affective touch. The mPFC, a key node of the ‘social brain’ (Frith *et al.*, 2003; Amodio and Frith, 2006; Frith and Frith, 2006; Mar, 2011; Sperduti *et al.*, 2011) is well known for its involvement in theory of mind, mindreading and mentalizing abilities (for reviews see Mar, 2011; Sperduti *et al.*, 2011), and is implicated in inferring other people’s intentions and mental states as well as attributing emotional states to others (Mar, 2011; Sperduti *et al.*, 2011; Schurz *et al.*, 2014). Previous neuroimaging evidence reported significant deactivation during the observation of touch (specifically any touch observation condition *vs* baseline) in bilateral mPFC (Ebisch *et al.*, 2011). On the other hand, a study by Gordon *et al.* (2013) reported that during CT-targeted gentle touch to the arm compared to palm, activation in right mPFC showed greater connectivity with left insula and amygdala, which may represent a coding of the social relevance and social reward of the tactile stimuli. In the current investigation, reduction in pleasantness ratings for other-directed touch upon disruption of mPFC might be linked to inaccuracies (or not being able to) in inferring and attributing pleasantness of touch for someone else (Stuss *et al.*, 2001). Nevertheless, and contrary to our expectation, inhibition of the mPFC was not causative of a reduction in pleasantness ratings specifically in the case of CT-targeted (slow) affective touch for another. In fact, the Bayes factor analysis provided more evidence for the null hypothesis that is the modulation of mPFC-cTBS on other-directed touch ratings was not specific for CT-optimal (i.e. slow) stroking speeds. Therefore, we suggest this brain region may be involved in the processing of affective touch when viewing someone else receiving touch and that the processing of CT-optimal touch occurs outside of mPFC. In addition, no significant correlations were observed between changes in pleasantness after mPFC-cTBS and varying levels of EDs symptoms, tactile distortions and touch experiences, nor with metacognitive interoception, which likely might be due to the relatively small size of our sample.

Our study offers insight into the functional role of the mPFC and S1 in shared representations of other- and self-directed interpersonal touch, nonetheless several limitations have been identified. Firstly, the videos used in the current investigation offer no contextual information which are important for touch pleasantness, such as visual/auditory cues regarding the touch giver (Macaluso and Driver, 2001; Taylor-Clarke *et al.*, 2002) and motivation and mood (Kalaska, 1994; Montoya and Sitges, 2006; Triscoli *et al.*, 2014). These are key features for the understanding how important touch is and how positive or negative it is (Ellingsen *et al.*, 2016). Furthermore, the relationship between the touch giver and receiver in the videos is important to highlight to participants, as this would control for the touch giver participants are imagining receiving the observed touch from. This way, participants would be able to fully embody the observed touch and imagine a scenario where they are receiving touch from a loved one or stranger It is well known indeed that romantic touch from a partner or touch received from a loved one is perceived as more pleasant than touch from a stranger (Suvilehto *et al.*, 2015; Bellard *et al.*, 2023), suggesting touch to be given from a stranger may impede negatively with their responses (Kreuder *et al.*, 2017). Therefore, contextual factors relating to touch pleasantness should be considered in light of the social relationship between touch giver and touch receiver. Moreover, results obtained for self- and other-directed touch should be handled cautiously due to the potential confound of the nature of the questions used for the two touch ratings, thus, no direct comparisons should be made. Whilst overall the two questions aimed at understanding how empathic vicarious experiences of touch might impact a participant’s ratings of observed touch (Haggarty *et al.*, 2023), they were measuring behavioural responses on two different scales (i.e. ‘desire/wanting to be touched’ for the self-directed ratings, and ‘overt’ evaluation of pleasantness for the other person receiving the touch). Accordingly, it might be possible that whilst for self-directed touch, participants’ ratings might relate more to the affective reaction to the hedonic evaluation of the rewarding tactile interaction, on the other hand, the other-directed touch ratings might correspond to the motivational value and incentive attributed to the rewarding tactile stimulus for touch received by others (Triscoli *et al.*, 2014). These two evaluative dimensions can under specific circumstances dissociate from one other, so that for example, after an aversive experience, explicit wanting and anticipatory pleasure of interpersonal touch are enhanced, without a corresponding change in the liking expressed during and after consumption (Massaccesi *et al.*, 2021).

In this study, we did not include a measure of perspective taking/mentalizing, for example the perspective-taking subscale of the interpersonal reactivity index (IRI; Davis, 1983), previously successfully used in demonstrating links between empathy and vicarious representations of touch (Gazzola *et al.*, 2006; Schaefer *et al.*, 2012; Bolognini *et al.*, 2013, 2014). Whilst a previous related investigation by Adler and Gillmeister (2019) did not find evidence to suggest a link between somatosensory physiological markers of vicarious touch and individuals’ perspective-taking abilities, it might be, as pointed by the two authors, that previously reported associations in the literature result from feedback from later cognitive processes rather than affecting S1 processing directly, a mechanism which might be instead housed in mPFC. Future studies might further elucidate the links between perspective taking abilities and shared representations of vicarious touch responses in key nodes of the ‘social brain’, including the mPFC.

Furthermore, it should be noted that the activation of S1 might be reliant on the level to which the individual resembles the observed body part as their own, as well as perspective, with specular (mirror-like) mapping in S1 for third person perspective occurring at a later stage of cortical somatosensory processing (Rigato *et al.*, 2019). In future, it could be useful to ensure participants fully embody the touch they are viewing, by using for example Virtual Reality, a form of technology previously successfully used in enhancing bodily ownership (de Jong *et al.*, 2017; Harjunen *et al.*, 2017; Della Longa *et al.*, 2022; Seinfeld *et al.*, 2022).

Finally, a further argument should be made on the localisation of S1 and mPFC and specifically in relation to the extent to which the targeted cortical regions were actually stimulated with TMS. Whilst the co-registration of coil placement with individual MRI images and the use of a real-time neuro-navigation system adopted in our study ensured precise anatomical targeting, yet it is still very possible that the stimulation of S1 by TMS has also affected nearby posterior parietal sites, including the motor cortex (Chan and Baker, 2015) if one considers TMS methodological limitations of spatial resolution and its indirect effects on connected areas (Tamè *et al.*, 2015). As for the localization of the mPFC, a recent review by Lieberman *et al.* (2019) suggests that whilst there is consistent evidence that the mPFC plays a causal role in social cognition (primarily observed for studies of emotion perception and trait judgments), it should be noted that, in the domain of TMS, these are mainly supported by investigations focusing on the dorsomedial portion of the PFC, in Brodmann area 9 (Lieberman *et al.*, 2019). The reason for this is that conventional, flat figure-8 rTMS coils are unable to reach deeper portions of the PFC, and therefore TMS is ill-suited to this scope. At present, we cannot provide any strong conclusions about the causative role of mPFC in vicarious affective touch, when considering its anatomical subdivisions. Future studies should focus on this question by employing more suitable methods, e.g. transcranial focused ultrasound, which offer several advantages over TMS methods including high spatial resolution and the ability to reach deep brain targets (Darmani *et al.*, 2022).

Our results provide supportive evidence that distinct vicarious social touch mechanisms exist to support simulations of bodily events when these are related to the self as compared to others. Specifically, we speculate that whereas right S1 may be crucial for the visuo-tactile mirroring and representation of touch self-relatedness, the right mPFC, a core node of the ‘social brain’ may be instead actively involved in representing tactile outcomes for the bodies of others. We also report preliminary evidence that visuo-tactile mirroring for self-directed touch in S1 is linked to individual differences in emotional awareness, thus paving the way for future investigations looking at associations between alteration of somatosensory cortex with difficulties in emotional awareness during interpersonal touch scenarios (Kanbara and Fukunaga, 2016). These TMS effects were not specific for CT-targeted (slow) affective touch, but rather they applied to all types of social touch. To conclude, our study challenges the causal role of the S1 and mPFC in vicarious affective touch and suggests that self *vs* other-directed vicarious touch responses might crucially depend on the specific involvement of key social networks in tactile interactions.

## Data Availability

The data that support the findings of this study are free and available to access in OSF https://osf.io/7guda/?view_only=fefce28d956341069d919bce3f396566.
